# Robust endoscopic image mosaicking via fusion of multimodal estimation

**DOI:** 10.1016/j.media.2022.102709

**Published:** 2023-02

**Authors:** Liang Li, Evangelos Mazomenos, James H. Chandler, Keith L. Obstein, Pietro Valdastri, Danail Stoyanov, Francisco Vasconcelos

**Affiliations:** a Wellcome/EPSRC Centre for Interventional and Surgical Sciences(WEISS) and Department of Computer Science, University College London, London, UK; b Storm Lab UK, School of Electronic, and Electrical Engineering, University of Leeds, Leeds LS2 9JT, UK; c Division of Gastroenterology, Hepatology, and Nutrition, Vanderbilt University Medical Center, Nashville, TN 37232, USA; d STORM Lab, Department of Mechanical Engineering, Vanderbilt University, Nashville, TN 37235, USA; e College of Control Science and Engineering, Zhejiang University, Hangzhou, 310027, China

**Keywords:** Medical image processing, Optical flow, Image mosaicking, Pose graph optimisation, Endoscopic image mosaicking

## Abstract

We propose an endoscopic image mosaicking algorithm that is robust to light conditioning changes, specular reflections, and feature-less scenes. These conditions are especially common in minimally invasive surgery where the light source moves with the camera to dynamically illuminate close range scenes. This makes it difficult for a single image registration method to robustly track camera motion and then generate consistent mosaics of the expanded surgical scene across different and heterogeneous environments. Instead of relying on one specialised feature extractor or image registration method, we propose to fuse different image registration algorithms according to their uncertainties, formulating the problem as affine pose graph optimisation. This allows to combine landmarks, dense intensity registration, and learning-based approaches in a single framework. To demonstrate our application we consider deep learning-based optical flow, hand-crafted features, and intensity-based registration, however, the framework is general and could take as input other sources of motion estimation, including other sensor modalities. We validate the performance of our approach on three datasets with very different characteristics to highlighting its generalisability, demonstrating the advantages of our proposed fusion framework. While each individual registration algorithm eventually fails drastically on certain surgical scenes, the fusion approach flexibly determines which algorithms to use and in which proportion to more robustly obtain consistent mosaics.

## Highlights

• We propose a robust endoscopic image mosaicking algorithm in surgical scenes.
• The framework fuses image data association algorithms based on based on their uncertainties.
• The fusion scheme is formulated in general form and can be extended to other problems.
• Generalisability is validated by extensive experiments in very different surgeries.
• The fusion approach is compared against the individual estimation approaches.

## Graphical abstract

**Figure:**
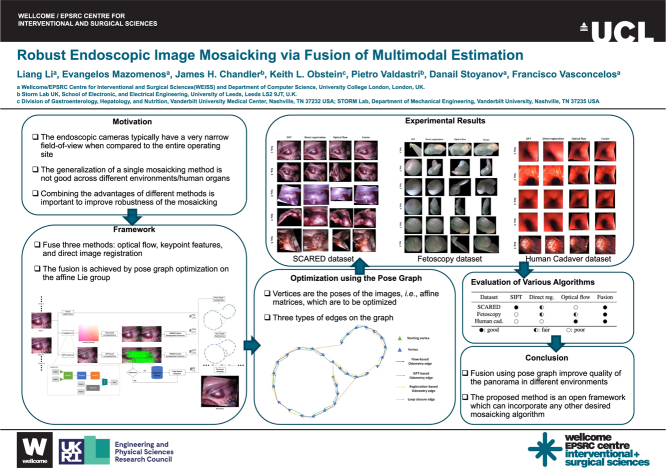

## 1 Introduction

Image mosaicking, or image stitching, is an established technique in computer vision that is now widely utilised in robotics and consumer products such as cell phones. In minimally invasive surgeries guided by a camera scope with a narrow field of view, mosaicking can generate an expanded view of operative site that can aid the surgeon in navigating instruments and planning the surgery (Kutarnia and Pedersen, 2015). Unlike very well established mosaicking applications involving indoor or outdoors scenes (Oliveira et al., 2015, Xu et al., 2020, Chon et al., 2007), mosaicking of endoscopic images has significantly increased challenges (Loewke et al., 2020, Richa et al., 2014, Loewke et al., 2010) that can take multiple forms that we now detail. The scene illumination is severely non-homogeneous as the only light source comes from the endoscopic camera itself and moves within the environment. Tissue and organs are very prone to saturated specular reflections that dynamically change with the camera motion (Wu and Su, 2017). Tissue can be dynamically occluded by blood or other artefacts (e.g. floating particles in fetoscopy) that have motion patterns inconsistent with the camera motion (Reeff et al., 2006). There are non-rigid tissue deformations caused by breathing, blood flow, or surgical instrument manipulation (Zhou and Jayender, 2021). The entire visualisation of the operative site can take a significant amount of time within the surgical workflow, and long-term mosaic consistency cannot be ignored (Li et al., 2021). Finally, the visual appearance of different environments/organs vary significantly, making feature extraction difficult to generalise. An algorithm that may work robustly in a narrowly defined environment will eventually degrade or fail when there are substantial changes in scene appearance (Bano et al., 2020). Addressing these challenges in a robust way is fundamental since an image mosaic can be rendered unusable with just a short number of poorly estimated image registrations.

Generally, image mosaicking consists of different sub-problems. The first one is the data association between common parts of the scene under different views (Huang and Netravali, 2002). The second is the estimation of a geometric transformation that is consistent with the data association and maps different views into a single mosaic image (Chum and Matas, 2012). These two sub-problems also be tackled simultaneously (*e.g.*, direct registration (Bartoli, 2008)). Finally, the image intensities of individual images need to be blended in a consistent and smooth mosaic (Tian and Shi, 2014). One can also consider the global optimisation of long-term mosaics as a separate sub-problem (Zhou and Jayender, 2021, Li et al., 2021). The first sub-problem of data association is the most challenging in surgical scenes and draws a significant amount of research attention. The most classic approach is to detect and extract image point features corresponding to unique landmarks in the scene and then match them across different views. This feature-based mosaicking approach (Milgram, 1975) has been investigated extensively in recent decades, using different well-known hand-crafted feature approaches such as Harris (Okumura et al., 2013), SIFT (Li et al., 2008), SURF (Rong et al., 2009), ORB (Chaudhari et al., 2017), and FAST (Wang et al., 2012). More recently, data-driven features that are learned by deep neural networks have been utilised for image mosaicking (Bano et al., 2020, Zhang et al., 2019). There are also mosaicking approaches that do not rely on feature extraction. Direct and dense pixel-based registration methods can be formulated as an iterative optimisation problem by maximising the similarity computed with mutual information (Miranda-Luna et al., 2008) or other photometric similarity/difference metrics (Levin et al., 2004, Konen et al., 2007). With the popularisation of deep learning in different problems, some end-to-end mosaicking algorithms based on deep learning regression of registration parameters (Bano et al., 2019, Nguyen et al., 2018) have been proposed.

There is also research focused on developing image mosaicking methods that are dedicated to deal with surgical scenes and its associated challenges. In Zhou and Jayender (2021), non-rigid Simultaneous Localization And Mapping (SLAM) is adopted to account for tissue deformation. Similarly in Loewke et al. (2010), deformation and cumulative errors are addressed with local and global alignment. In Soper et al. (2012), structure-from-motion with bundle adjustment is utilised to reduce cumulative errors when generating the mosaic. In Gong et al. (2021), the non-rigid deformation is estimated with a parametric free form deformation model. By reviewing the literature, it is clear that most of the existing mosaicking algorithms in this domain focus on estimating the deformation or reducing cumulative errors. However, the problems of inconsistent light conditions and environment changes have not been analysed in detail. These can happen frequently when generalising a method to work robustly on different cases, where the camera scope and light source may have settings, or a different patient may have anatomy structures with different appearance. With these challenges in mind, this paper aims to solve the problem of robustness in image data association for mosaicking of surgical scenes. Instead of choosing between point feature extraction, optimisation of photometric alignment, or a deep learning approach, we propose to fuse multi-modal estimation to bring the best of each method. Our fusion framework is agnostic to the data source and can be easily generalised to other contexts. In this paper we consider as an exemplary case the fusion of three sources: optical flow, hand-crafted (scale-invariant feature transform) SIFT features, and direct photometric registration. The considered optical flow method is the end-to-end deep neural network FlowNet2.0 (Ilg et al., 2017). After data association between different frames, the geometric alignment between different views is modelled as a homography linear mapping, and approximated as an affine transformation. For both hand-crafted features and optical flow, (random sample consensus) RANSAC is used to filter out outliers prior to registration estimation. The core of our proposed method is to take all the available and competing motion estimation approaches as inputs to a pose graph optimisation framework. Considering different camera views as graph nodes, up to three edges representing different motion estimations will link them. The optimal graph state is computed using the Levenberg–Marquardt (L–M) algorithm on the affine Lie group. The experimental results show that the proposed fusion-based image mosaicking algorithm outperforms keypoint feature-based, dense registration, and end-to-end algorithms in terms of robustness, consistency and generalisation to different datasets. Therefore, the contributions of this paper are threefold:

1. We propose a framework to fuse different image data association algorithms based on their uncertainties for endoscopic mosaicking. The proposed method improves robustness and adaptability across various types of surgical scenes.
2. The proposed fusion scheme is formulated in general form and is not constrained to any specific estimation sources, nor to the type of surgery. It can easily be extended to other problems involving multi-modal estimation and/or data sources.
3. Extensive experiments in significantly different surgeries are carried out to validate the generalisability of the proposed method. We test more than ten sequences of ex-vivo laparoscopic video from the publicly available SCARED dataset (Allan et al., 2021), a publicly available fetoscopic surgery dataset (Bano et al., 2019), and also cadaver sequences captured with the Bellowscope robotic gastric endoscopy platform (Chandler et al., 2020, Garbin et al., 2018, Garbin et al., 2019). The fusion approach is compared against the individual estimation approaches, *i.e.*, SIFT-based, direct registration-based, end-to-end deep learning-based mosaicking.

The remainder of this paper is organised as follows. Section 2 gives a review of the related work. Section 3 introduces the formulations in correspondence matching and homography estimation, and details our proposed fusion-based mosaicking framework. Section 4 presents and discusses the experimental results. We finally conclude the paper and provide some remarks on future work in Section 5.

## 2 Related work

While image mosaicking is a problem with a wide variety of well established application domains, medical imaging has its own dedicated challenges. Therefore, in this section we concentrate on methods directly applied to surgical data. The algorithms can be classified into three categories: feature-based, direct, and deep learning-based.

Feature-based mosaicking has been studied for decades in the context of medical imaging. Early work can be found in Can et al., 2002b, Can et al., 2002a, where the edges of vascular centrelines in human retina are used as features. To speed up the mosaicking, a hierarchical registration algorithm was adopted. In Lee and Bajcsy (2005), the centroids of vascular regions were selected as features for image registration and mosaicking, and a normalised correlation-based registration algorithm is used to estimate affine transformations. In Bergen et al. (2009), the authors used corner-like features and Kanade–Lucas–Tomasi tracker (KLT) to track features in subsequent frames rather than using feature matching. With the development of abstract feature extraction in the community of computer vision, some state-of-the-art feature descriptors were also utilised for mosaicking of medical images. Despite its relatively old age, SIFT (Lowe, 1999) is one of the most widely utilised feature descriptors (Daga et al., 2016, Richa et al., 2014, Jalili et al., 2020). Other popular handcrafted approaches include improved modifications of SIFT (Yu et al., 2015, Li et al., 2017, Gupta et al., 2016) and (speeded up robust features) SURF (Bay et al., 2006, Reeff et al., 2006). (oriented FAST and rotated BRIEF) ORB features have also been utilised for mosaicking in the context of robotic endomicroscopy (Rosa et al., 2018). Notably, here the authors fuse robot movement information with image registration to produce more robust estimation. Independently of utilising different image feature descriptors, we also note that different feature matching algorithms can also be considered (Viergever et al., 2016, Sotiras et al., 2013).

Unlike feature-based methods, direct registration aims at using the information of all image pixels (Baum et al., 2021). In Ogien et al. (2020), normalised cross-correlation (NCC) was used to maximise similarity between registered images. Furthermore, in Capek and Krekule (1999), three similarity-based methods were studied, the sum of absolute valued differences (SAVD), NCC, and the mutual information function (MIF). It was reported that SAVD performs best in terms of computational efficiency, NCC is more robust to uncorrelated stochastic noise, and MIF outperforms the other two in terms of nonlinear corruption of the intensity scales of the image. In Seshamani et al. (2009), the sum of squared differences (SSD) was used to measure difference of the two images in terms of their intensities. The first-order Taylor linearisation was utilised to minimise the SSD. Moreover, bundle adjustment that minimises the total point re-projection error was adopted for global optimisation. In Seshamani et al. (2006), the authors adopted a two-step optimisation algorithm. In the first stage, an initial estimate of 2D translation is computed by performing a brute-force search to maximise normalised cross-correlation between images; In the second stage, a local continuous finetune is applied by minimising intensity difference of the two images. In Peter et al. (2018), a pixel-wise image gradient alignment was adopted to highlight vessel-like structures. In Richa et al. (2014), an SSD-like function was used to minimise the intensity dissimilarities between two images. And a non-rigid illumination compensation fine-tuning was adopted to model local variations. Some studies try to combine feature-based and direct methods to take advantage of both (Richa et al., 2012).

Deep learning based methods have drawn more attention in recent years. Some researchers made efforts to utilise deep learning either in feature extraction or end-to-end transformation regression. In Bano et al. (2020), U-Net (Ronneberger et al., 2015) was used to segment vessels in fetoscopic images. Then, a direct image registration based on the output probability map of the neural network was proposed to estimate the homography matrix. In Bano et al. (2020), a deep image homography with controlled data augmentation was proposed to estimate homography between the two input images directly. To the best of the authors’ knowledge, there is no study addressing the robustness and generalisation challenges across different environments in the context of surgical video mosaicking. This is an important problem that can arise in different domains, including GI endoscopy, fetoscopic surgery, and laparoscopy. This paper proposes to solve robustness challenges and generalisability by fusing multimodal estimation within an affine pose graph framework.

**Fig. 1:**
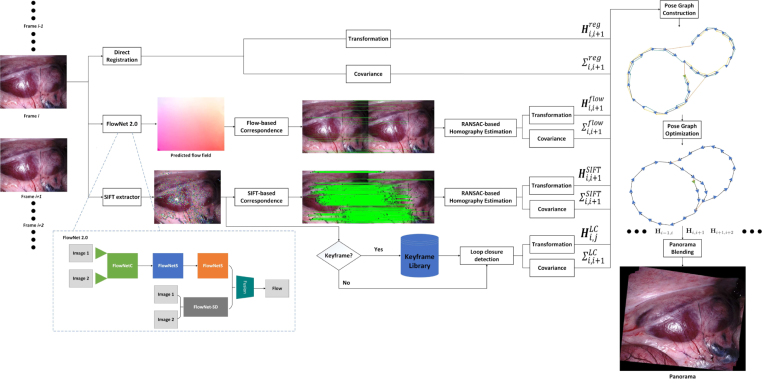
The diagram of the proposed method. There are three component homography estimation algorithms, *i.e.*, SIFT-based, direct registration-based, and the optical flow-based. The pose graph is constructed based on the three estimation sources with their own uncertainties respectively. The optimal state is obtained by optimising the cost function in the affine Lie group. Finally, the panorama can be generated with the optimal homography matrices.

## 3 Approach

This section presents the proposed algorithm for surgical video mosaicking. The diagram of the proposed algorithm is presented in Fig. 1, displaying its several different components. It contains the three baseline data association methods (optical flow, handcrafted features, direct registration). Additionally, a loop closure detection source is included based on storing handcrafted features in keyframes. The fusion of multimodal results is achieved with pose graph optimisation, and finally image stitching and blending is performed based on the optimised graph. While direct registration performs data association and registration simultaneously, both handcrafted features and optical flow only perform the data association. Thus they require a second step to estimate homography transformations via 4-point linear estimation within a RANSAC robust estimator. We fuse all three methods together with pose graph optimisation to make the estimation more robust. Finally, all the images can be stitched together with respect to the middle frame within the sequence.

### 3.1 Optical flow-based correspondence

While both direct pixel-based registration and feature-based registration are classic approaches that have been extensively described in the previous works, image registration based on general optical flow is less common, especially with more recent deep learning methods, and therefore we provide here a more detailed account.

Optical flow measures displacement of pixels in two images. It is computed based on the assumption that intensity of the same object is constant in the consecutive frames, *i.e.*:

(1) I(x,y,t)=I(x+dx,y+dy,t+dt)

where I(x,y,t) denotes the intensity of pixel (x,y) at time t. Currently, there are several ways to compute the optical flow. The first type is the gradient-based method that includes Lucas-Kanade and Horn–Schunck. It assumes that the optical flow is smooth on the entire image. While in our case, specularities may degenerate the computation. The second type is the matching-based method that starts from sparse feature correspondences and interpolates a flow field for every pixel. This is not suitable in our case as it would be redundant with feature-based registration and fail in the same cases. The third type is the energy minimisation approach that uses the dense information of the whole image. Again, this approach may be affected by inconsistent specular reflections. More recently, the energy minimisation methods can be optimised on training data with a deep neural network. In this paper, we use the deep learning-based method FlowNet2.0 due to its state-of-the-art performance. It has three types of components: FlowNetSimple (FlowNetS), FlowNetCorrelation (FlowNetC) which are proposed in FlowNet (Dosovitskiy et al., 2015), and FlowNet-Small-Displacement (FlowNet-SD) which is finetuned on a stack of FlowNetS and FlowNetC. Input of FlowNetS is a stack of two images, and the network architecture follows an encoder–decoder framework. It has six convolutional layers, four deconvolutional layers and finally a bilinear upsampling to lift the prediction map to full image resolution.

In order to make the network more efficient at catching salient features, FlowNetC first adopts two independent, yet identical processing streams for the two images separately. The two embeddings are then combined with a correlation layer that aids the following network to find correspondence. Given two patches centred at $x_{1}$ in the first feature map $f_{1}$ and $x_{2}$ in the second feature map $f_{2}$, the correlation layer is:

(2) $$c\left(x_{1},x_{2}\right)=\underset{o\in \left[-k,k\right]\times \left[-k,k\right]}{\sum }\langle f_{1}\left(x_{1}+o\right),f_{2}\left(x_{2},o\right)\rangle$$

**Fig. 2:**
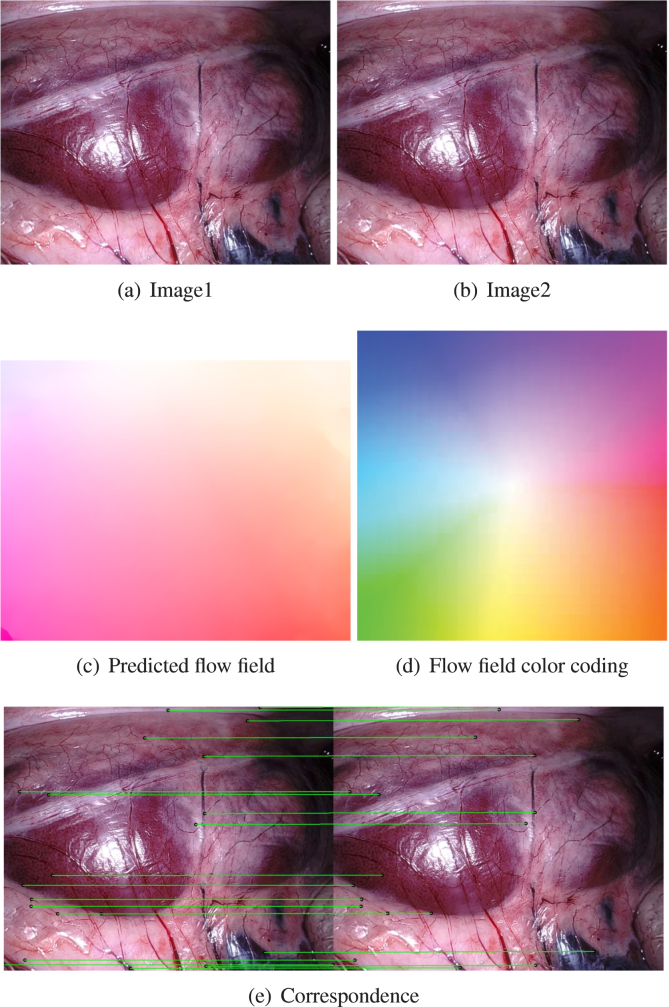
An example of the results of optical flow prediction and correspondence establishment. (a) and (b) show the two input images, and (c) is the predicted flow field by the Flownet2.0, where the colour coding scheme is shown in (d). The correspondence can be established using Eq. (3). In theory, the correspondence is very dense as correspondence for most pixels can be computed except ones close to the image border. Only a small portion of the correspondence is presented in (e) for a better visualisation.

Note that it is identical to one-step convolution with the kernel as data from another feature map rather than the filter. It limits the maximal displacement k to within only the local neighbourhood to reduce the computation. After the correlation layer, FlowNetC adopts the FlowNetS to predict the optical flow. It was reported in Ilg et al. (2017) that FlowNetC outperforms FlowNetS if training under the same condition. FlowNet-SD is based on a stack of one FlowNetC and two FlowNetS (FlowNet-CSS). FlowNet-SD deepens the network with multiple layers with 3×3 kernels at the beginning of the network and is trained on dataset with small displacement. Finally, FlowNet2.0 fuses FlowNet-CSS and FlowNet-SD to give the predicted flow field to full resolution as the input image. One benefit of FlowNet2.0 is its generalisation, *i.e.*, we do not need to re-train it on surgical data for our mosaicking task. The network is trained from simple to more realistic datasets, *i.e.*, from the FlyingChairs synthetic dataset, to the FlyingThings3D synthetic dataset (Mayer et al., 2016), and finally on the KITTI real video dataset (Geiger et al., 2012). Examples of the predicted flow field on the endoscopic data are shown in Fig. 2(e).

The correspondence between the two images can be obtained by the flow field:

(3) $$\left[\begin{array}{c} u\\ v \end{array}\right]=\left[\begin{array}{c} u^{\prime }\\ v^{\prime } \end{array}\right]+\left[\begin{array}{c} o_{x}\\ o_{y} \end{array}\right]$$

where ${\left[\begin{array}{cc} u & v \end{array}\right]}^{⊤}$ is the position of the keypoint in the target image, and ${\left[\begin{array}{cc} u^{\prime } & v^{\prime } \end{array}\right]}^{⊤}$is the corresponding keypoint in the source image, ${\left[\begin{array}{cc} o_{x} & o_{y} \end{array}\right]}^{⊤}$ is the value of optical flow. An example of the correspondence estimation based on the optical flow is shown in Fig. 2(e). From this point onwards, pairwise point correspondences between two frames are established and the remaining registration pipeline is identical to estimation with sparse feature correspondences, *i.e.*, SIFT in this paper.

### 3.2 Homography estimation

Both optical flow and SIFT provide pairwise point correspondences, dense and sparse respectively, and estimating a homography registration can be made identical for both methods. On the other hand, the computation of homographies based on direct pixel-based image registration is jointly done with data association. In this subsection, we first derive the correspondence-based homography estimation, then the direct registration-based homography estimation. The transformation between correspondence pairs can be modelled as:

(4) $$\left[\begin{array}{c} u\\ v\\ 1 \end{array}\right]=s\cdot H\cdot \left[\begin{array}{c} u^{\prime }\\ v^{\prime }\\ 1 \end{array}\right]=s\left[\begin{array}{ccc} h_{11} & h_{12} & h_{13}\\ h_{21} & h_{22} & h_{23}\\ h_{31} & h_{32} & h_{33} \end{array}\right]\left[\begin{array}{c} u^{\prime }\\ v^{\prime }\\ 1 \end{array}\right]$$

where H is a 3×3 homography matrix, *i.e.*, $h_{33}=1$, and s is the scaling factor. In theory, the transformation is projective with a scaling factor. While as indicated in Bano et al., 2020, Peter et al., 2018, Li et al., 2021, approximating it with affine transformation gives more stable results for the endoscopic mosaicking. Thus, we follow this conclusion and assume s⋅H to be affine, *i.e.*, s=1, $h_{31}=h_{32}=0$, $\left[\begin{array}{cc} h_{11} & h_{12}\\ h_{21} & h_{22} \end{array}\right]$ is an arbitary non-singular matrix, and $\left[\begin{array}{c} h_{13}\\ h_{23} \end{array}\right]$ is the translation vector. The correspondence pair can be either from optical flow or SIFT. Every correspondence pair gives two constraints:

(5) $$u=\frac{h_{11}u^{\prime }+h_{12}v^{\prime }+h_{13}}{h_{31}u^{\prime }+h_{32}v^{\prime }+1}$$

$$v=\frac{h_{21}u^{\prime }+h_{22}v^{\prime }+h_{23}}{h_{31}u^{\prime }+h_{32}v^{\prime }+1}$$

If there are n pairs of correspondence, a linear matrix equation can be obtained:

(6) $$\underset{\underset{2n\times 9}{A}}{\left[\begin{array}{ccccccccc} u_{1}^{\prime } & v_{1}^{\prime } & 1 & 0 & 0 & 0 & -u_{1}u_{1}^{\prime } & -u_{1}v_{1}^{\prime } & -u_{1}\\ 0 & 0 & 0 & u_{1}^{\prime } & v_{1}^{\prime } & 1 & -v_{1}u_{1}^{\prime } & -v_{1}v_{1}^{\prime } & -v_{1}\\ \; & \; & \; & \; & \vdots & \vdots & \; & \; & \;\\ u_{n}^{\prime } & v_{n}^{\prime } & 1 & 0 & 0 & 0 & -u_{n}u_{n}^{\prime } & -u_{n}v_{n}^{\prime } & -u_{n}\\ 0 & 0 & 0 & u_{n}^{\prime } & v_{n}^{\prime } & 1 & -v_{n}u_{n}^{\prime } & -v_{n}v_{n}^{\prime } & -v_{n} \end{array}\right]}\underset{\underset{9}{h}}{\left[\begin{array}{c} h_{11}\\ h_{12}\\ h_{13}\\ h_{21}\\ h_{22}\\ h_{23}\\ h_{31}\\ h_{32}\\ 1 \end{array}\right]}=\underset{\underset{2n}{0}}{\left[\begin{array}{c} 0\\ 0\\ \vdots \\ 0\\ 0 \end{array}\right]}$$

So, if we have four pairs of correspondence, Eq. (6) can be solved. If there are more than four pairs of correspondence, the optimal solution can be obtained by minimising

(7) $$\arg\underset{h}{\min}\|Ah-0\|=\arg\underset{h}{\min}\|Ah\|$$

As we have the result of optical flow for every pixel, we can have w×h pairs of correspondence in theory, where w, and h are the width and height of the image respectively. Even for the SIFT, there may be hundreds of thousands pairs of corresponding points. Solving the problem by minimising Eq. (7) has two problems: First, computation of the optimisation problem will be very high; Second, the outliers and noise of the correspondence estimation may deteriorate the estimated homography matrix. So in this paper, we use RANSAC with the 4-point method to identify inliers and outliers. Here, the recognition of inliers and outliers is based on the distance in pixels between a point in one image and its re-projected correspondence from the other image through the transformation $\overset{ˆ}{H}$. And ɛ is the threshold set by the user to identify outliers in the flow field.

The homography matrix can be also estimated using direct image registration with the photometric loss:

(8) $$L\left(H_{i,i+1}\right)=\|I_{i}-T\left(I_{i+1},H_{i,i+1}\right)\|$$

where the function $T\left(I_{i+1},H_{i,i+1}\right)$ warps the image $I_{i+1}$ with transformation. By transforming the image $I_{i+1}$ into a new position using $H_{i,i+1}$, we can obtain $I_{i+1}$ in its new position and view as ${\overset{\sim }{I}}_{i+1}=T\left(I_{i+1},H_{i,i+1}\right)$. The difference of Image $I_{i}$ and ${\overset{\sim }{I}}_{i+1}$ can be computed with their L2 norm. The optimal transformation matrix can be obtained by minimising the loss:

(9) $$H_{i,i+1}^{reg}=\arg\underset{H_{i,i+1}}{\min}L\left(H_{i,i+1}\right)$$

**Fig. 3:**
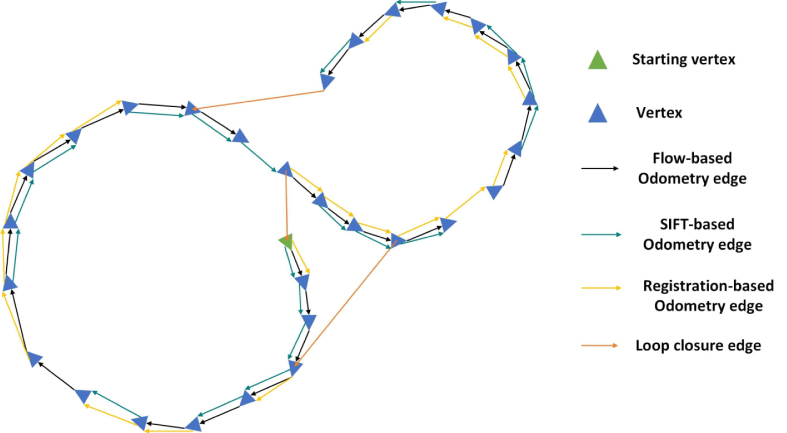
An illustration of the pose graph that is constructed using the optical flow, SIFT, direct registration, and loop closure detection. The nodes are denoted in blue triangles. And the different types of edges are denoted in lines with different colours.

**Fig. 4:**
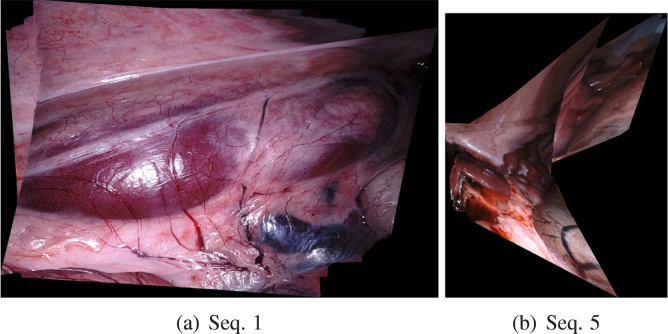
Examples of mosaicking directly obtained from using the robot kinematics, extracted from seq. 1 (a) and seq. 5 (b) of the SCARED dataset. The kinematics are not accurate enough to generate mosaics.

**Table 1:** Number of frames of the sequences for experiment.

Dataset	Sequence umber	Number of frames
SCARED	Seq. 1	196
	Seq. 2	279
	Seq. 3	87
	Seq. 4	447
	Seq. 5	347

Fetoscopy	Seq. 1	400
	Seq. 2	300
	Seq. 3	150
	Seq. 5	200
	Seq. 6	200

Human cadaver	Seq. 1	30
	Seq. 2	51
	Seq. 3	20
	Seq. 4	20
	Seq. 5	100

**Fig. 5:**
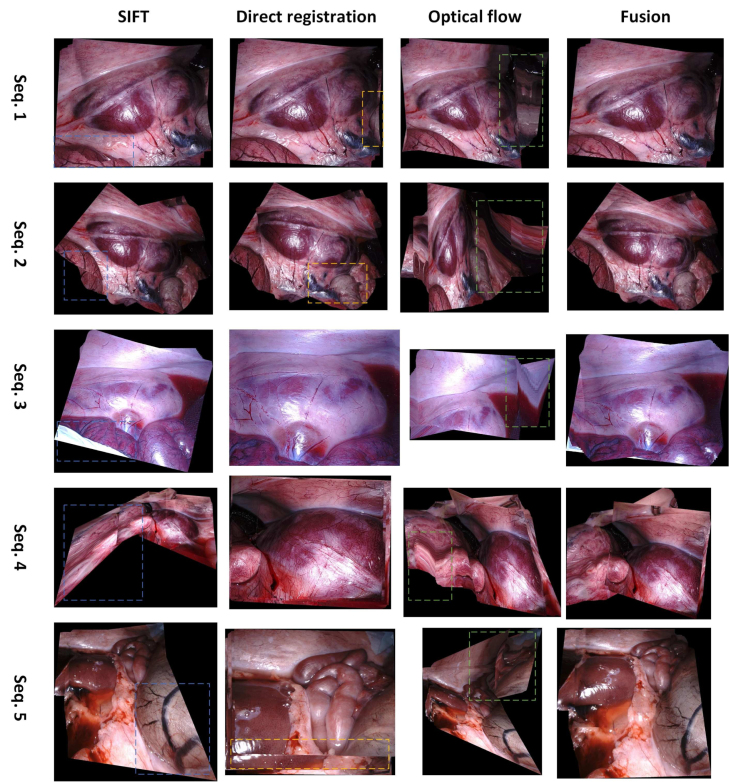
Results on the SCARED dataset. Mosaicking results for five sequences are presented from the first to the last row. The SIFT, direct registration, optical flow, and fusion-based mosaicking are presented from the first to the fourth column. The problematic parts of the panorama are denoted in blue, orange, and green rectangles from the first to the third column. The fusion-based mosaicking can correct them and combine advantages of the component methods to give high-quality panoramas.

**Fig. 6:**
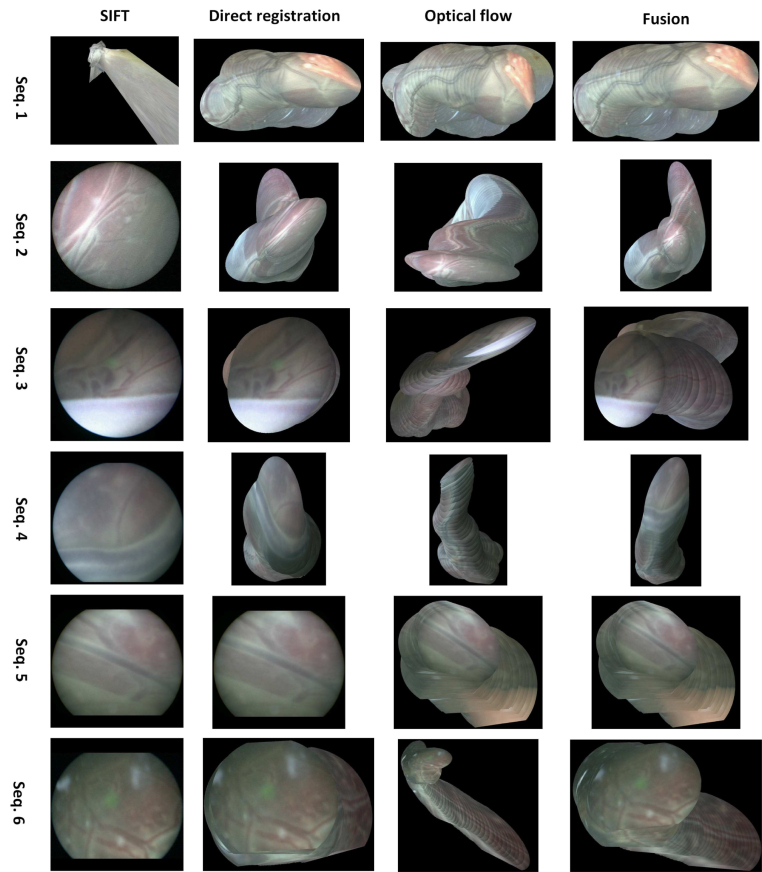
Results on the fetoscopy dataset. Mosaicking results for six sequences are presented from the first to the last row. The SIFT, direct registration, optical flow, and fusion-based mosaicking are presented from the first to the fourth column. SIFT-based method fails to work on this dataset due to the texture-less background and difficulty to extract enough features. The fusion-based method fuses results of the direct registration-based and optical flow-based homography estimation, and can combine the advantages of both methods to generate better panoramas.

**Fig. 7:**
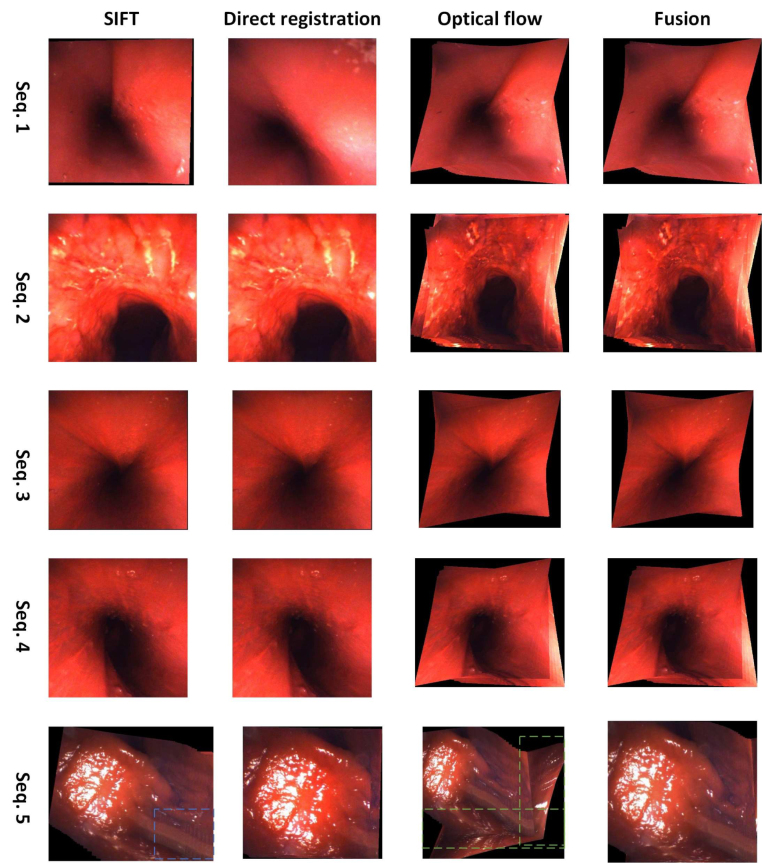
Results on the human cadaver dataset. Mosaicking results for five sequences are presented from the first to the last row. The SIFT, direct registration, optical flow, and fusion-based mosaicking are presented from the first to the fourth column. From the first to the fourth sequence, only the optical flow works among the three component methods. And the result of fusion is same as that of optical-flow mosaicking. For the fifth sequence, the fusion-based method fuses the results of SIFT-based and optical flow-based homography estimation using the affine pose graph, to yield a more consistent panorama.

**Fig. 8:**
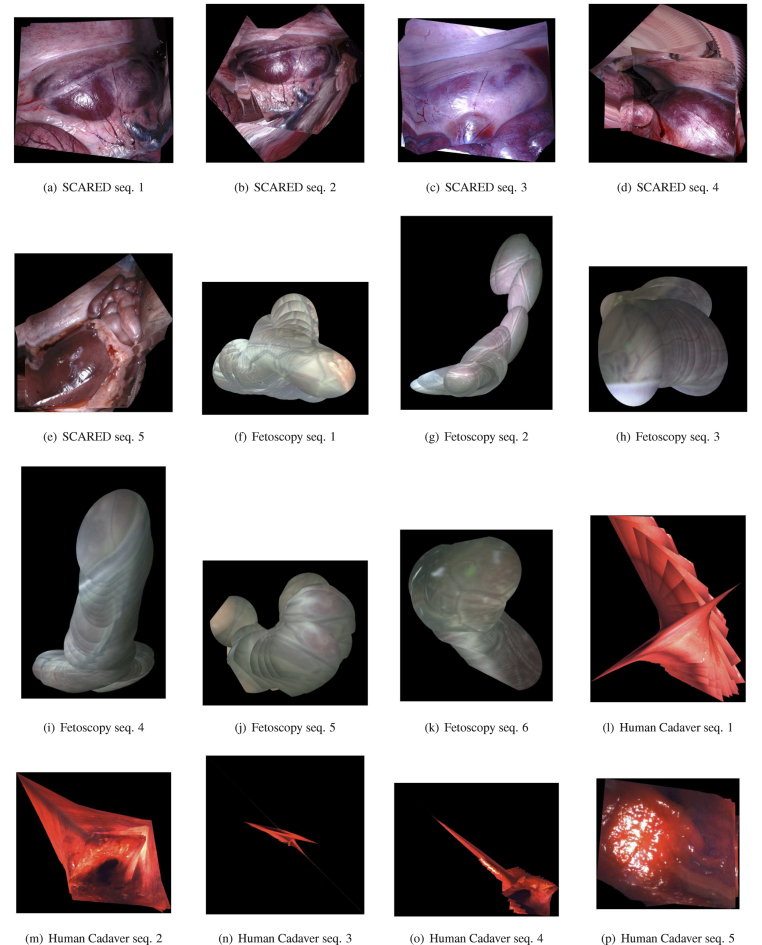
Mosaics generated by simple mean fusion of the SIFT-based, direct registration-based, and the optical flow-based estimation.

**Fig. 9:**
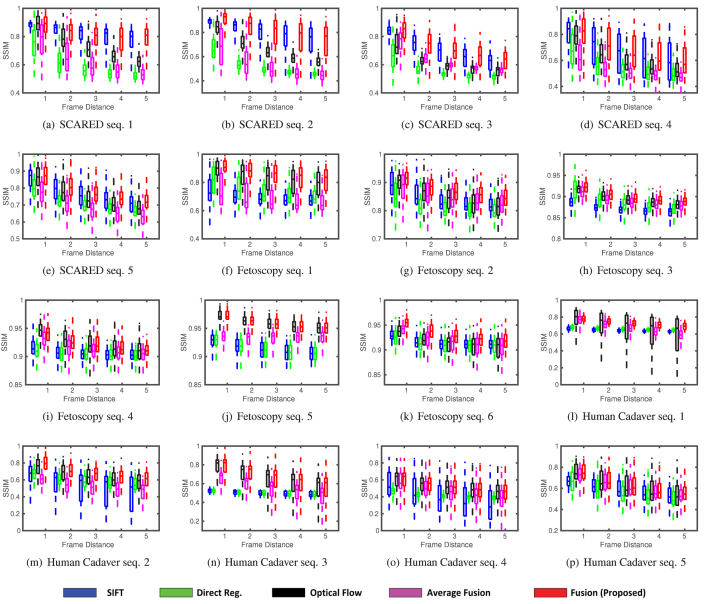
SSIM between overlapping registered frames with distance between 1 (consecutive) and 5. Each boxplot shows SSIM results of all frame pairs in a video with specified distance. Lower values denote poorer methods.

**Fig. 10:**
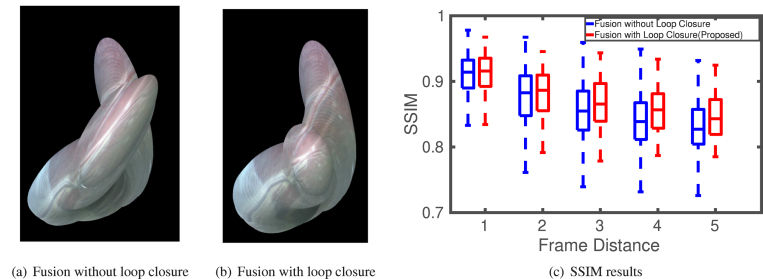
A comparison of mosaicking generated by fusion with and without loop closure on sequence 2 of the fetoscopy dataset.

The optimisation of Eq. (9) is based on a standard pyramidal Lucas–Kanade registration framework that minimises the least-square difference (photometric loss) between a fixed frame $I_{i}$ and a warped moving image ${\overset{\sim }{I}}_{i+1}$. This optimisation problem in Eq. (9) can be solved with the L–M iterative algorithm in an iterative way.

### 3.3 Fusion of multimodal estimations

For every pair of consecutive images, there are three possible estimated transformation matrices, *i.e.*, $H_{i,i+1}^{flow}$, $H_{i,i+1}^{SIFT}$, and $H_{i,i+1}^{reg}$. A more robust estimation result can be obtained by fusing the three estimation sources. Inspired by the SLAM literature in mobile robotics, we perform the fusion via pose graph optimisation. The graph to be optimised can be constructed as G={V,E}, where $V=\left\{x_{1},x_{2},\ldots ,x_{n}\right\}$ is the set of vertices and $E=\left\{z_{1,2},z_{2,3},\ldots ,z^{lc}\right\}$ is the set of edges. Both x and z are affine matrices, and z are the estimated transformation matrices $H^{flow}$, *etc*. An illustration of the pose graph is shown in Fig. 3. Any vertex $x_{i}$ in the graph represents the transformation of the ith image with respect to the anchor (first) image, and they constitute the state to be estimated (optimised). The edges define constraints between pairs of vertices, which can be provided by the affine homography estimations obtained from optical flow, SIFT-correspondences, and direct registration.

Additionally, edges can also be loop closure constraints, i.e. registration of non-consecutive frames when a scene is revisited. Loop closure detection is based on SIFT keypoint features extracted from a set of key frames. The first frame in the sequence is always a key frame. If the current movement with respect to the latest key frame is larger than either a distance or a time threshold, the current frame is defined as a new key frame. SIFT features of the key frames are stored using bag-of-words. The similarity between a new frame and every other key frame will be computed to check if the camera revisits previous scenes. Every estimated transformation matrix is associated with a covariance matrix $\Sigma_{i,i+1}$ representing how certain the estimation is. The covariance of the flow-based transformation $\Sigma_{i,i+1}^{flow}$ is computed based on the ratio of inliers. The covariance of the SIFT-based transformation $\Sigma_{i,i+1}^{SIFT}$ is jointly based on ratio of inliers and number of features. The covariance of the direct registration-based transformation $\Sigma_{i,i+1}^{reg}$ is based on the finally minimal photometric loss. Then we can define the cost function of the pose graph as:

(10) $$f=\underset{i,j\in C}{\sum }e_{ij}{\left(x_{i},x_{j},z_{i,j}\right)}^{⊤}\Omega_{ij}e\left(x_{i},x_{j},z_{i,j}\right)=\underset{i,j\in C^{flow}}{\sum }e_{ij}{\left(x_{i},x_{j},z_{i,j}^{flow}\right)}^{⊤}\Omega_{ij}^{flow}e\left(x_{i},x_{j},z_{i,j}^{flow}\right)+\;\underset{i,j\in C^{SIFT}}{\sum }e_{ij}{\left(x_{i},x_{j},z_{i,j}^{SIFT}\right)}^{⊤}\Omega_{ij}^{SIFT}e\left(x_{i},x_{j},z_{i,j}^{SIFT}\right)+\;\underset{i,j\in C^{reg}}{\sum }e_{ij}{\left(x_{i},x_{j},z_{i,j}^{reg}\right)}^{⊤}\Omega_{ij}^{reg}e\left(x_{i},x_{j},z_{i,j}^{reg}\right)$$

where C is set of all the edges including odometry edges (j=i+1) and loop closure edges (j≠i+1), and the function e measures errors between the vertices and constraints by the edges. Ω is the information matrix, *i.e.*, the inverse of the covariance matrix $\Omega =\Sigma^{-1}$. The covariance of an edge is decided based on the residual or number of correspondences of the two images. For the SIFT-based method, its covariance $\Sigma^{SIFT}$ is inversely proportional to the number of established pairwise point correspondences. For the optical flow-based method, the covariance $\Sigma^{flow}$ is inversely proportional to the number of RANSAC inliers as described in Section 3.2. For the registration-based method, the covariance $\Sigma^{reg}$ is directly proportional to the photometric residual after optimisation. In this paper, we set the information matrix as follows: if the number of inliers or correspondences is $N_{inl}$ for the SIFT-based or optical flow-based method, then the first four diagonal elements of the information matrix is set as $\Omega_{\left(1,1\right)}=\Omega_{\left(1,1\right)}=\cdots =\Omega_{\left(4,4\right)}=100\times N_{inl}$, the last two diagonal elements are set as $\Omega_{5,5}=\Omega_{6,6}=N_{inl}$. If the residual for the direct registration-based method is $e_{res}$, then the first four diagonal elements are set as $\frac{100}{e_{res}}$, and the last two diagonal elements are $\frac{1}{e_{res}}$. If there is not enough inliers/correspondences, the residual is too large, or the output of the RANSAC algorithm is an identity matrix, then we set all elements of information matrix as zero Ω=0, which means we treat the estimation as a failure and do not consider the constraint provided by this edge. SIFT may also have loop closures to measure errors between non-consecutive vertices. Note that there may exist better information matrix configuration strategies, we left it to be explored in our future work. The error function e needs to be converted from the 3×3 affine matrix to a vector to compute and minimise the loss. Following our previous work (Li et al., 2021), the vectorisation is based on the Lie group theory, *i.e.*, from element on affine Lie group to its corresponding Lie algebra and then the vector space. And update of the state is wrapping from the vector space to Lie group. For a detailed elaboration please refer to Li et al. (2021), while in this paper, the key procedures are introduced briefly. We have $e\left(x_{i},x_{j},z_{i,j}\right)=\log{\left(z_{i,j}^{-1}x_{i}^{-1}x_{j}\right)}^{\vee }$ using the logarithm map. Updating it with a small perturbation ξ in Lie algebra leads to e(xexp(ξ))≃e(x)+Jξ, where we take the first-order Taylor approximation and J is the Jacobian matrix from affine Lie group to vector space which can be computed by numerical method. The cost function on the updated state is

(11) $$f\left(x\exp\left(\xi \right)\right)≃f\left(x\right)+2\underset{b^{⊤}}{\underset{︸}{\underset{i,j\in C}{\sum }e{\left(x_{i},x_{j},z_{ij}\right)}^{⊤}\Omega_{ij}J_{ij}}}\xi +\;\xi^{⊤}\underset{K}{\underset{︸}{\underset{i,j\in C}{\sum }J_{ij}^{⊤}\Omega_{ij}J_{ij}}}\xi =f\left(x\right)+2b^{⊤}\xi +\xi^{⊤}K\xi$$

To make the update driven to the optimal value, we need to make the differential of the cost function equal to zero, *i.e.*, f(xexp(ξ))−f(x)=0. The differential of equation (11) with respect to ξ is Kξ+b=0, which is linear. To improve the convergence, we adopt L–M algorithm here by incorporating a damping factor λ as $\left(K+\lambda I\right)\xi^{*}+b=0$. Then the state can be updated in this step as:

(12) $$x^{*}=x\exp\left(\xi^{*\wedge }\right)$$

The procedures from Eq. (11) to Eq. (12) iterate to update the state until convergence.

**Figure:**
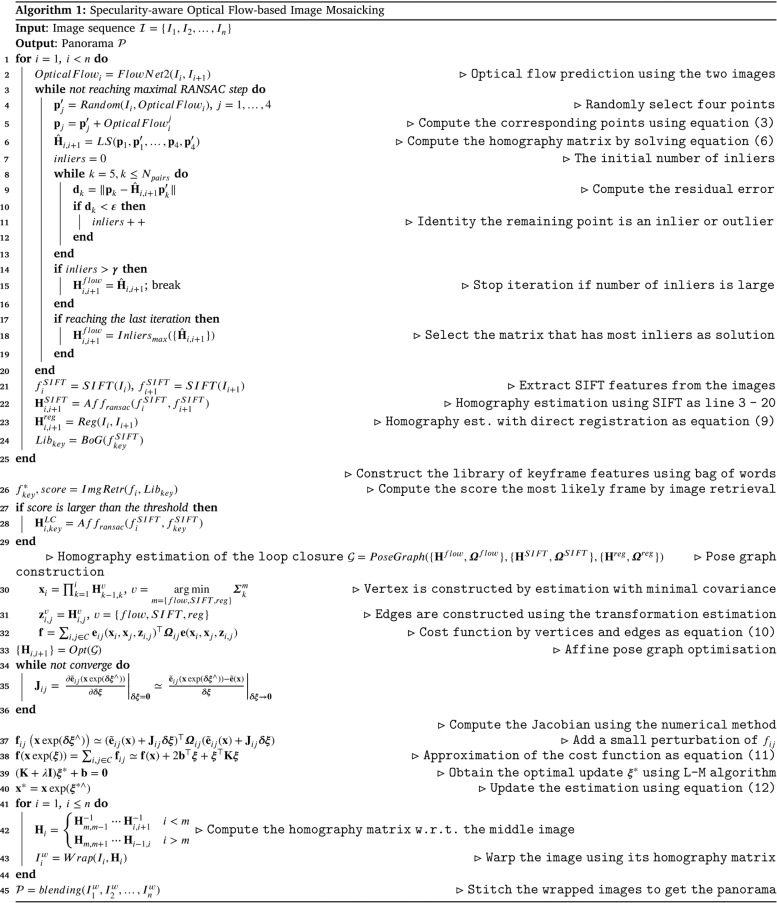

### 3.4 Panorama blending

Each image is attached with its own homography matrix with respect to its former image denoted as $\left\{I_{i},H_{i-1,i}\right\},i=1,2,\ldots ,n$, where n is the number of images and $H_{0,1}=I$ is the identity. To make a better visualisation, we set the transformation of the middle image ($\frac{n}{2}$ if n is an even number, $\frac{n+1}{2}$ otherwise) as the identity. Then every image can obtain its new transformation with respect to the middle image by matrix multiplication and inverse. For convenience, we use $H_{i}$ to denote transformation of i-th image. First, we need to compute the resolution of the panorama. Every image is warped to its position using the affiliated homography matrix $H_{i}$. The coordinates of four corners of the panorama can be obtained with minimal and maximal corners in two directions of all the wrapped images. In this way, we can create a blank mask that has the same size of the panorama. Then, for the first warped image, it can be fit into the mask directly. From the second image, only the mask’s pixels that are still blank will be substituted with the pixels of the wrapped image. The proposed algorithm is summarised in Algorithm 1.

## 4 Experiments

In this section, experiments on various endoscopic datasets and comparison with state-of-the-art baselines will be presented. We test on three datasets: The first one is the Stereo Correspondence and Reconstruction of Endoscopic Data (SCARED) dataset (Allan et al., 2021). The utilised sequences are from its training data: Seq. 1 ← dataset1/keyframe1, Seq. 2 ← dataset1/keyframe2, Seq. 3 ← dataset2/keyframe1, Seq. 4 ← dataset3/keyframe3, Seq. 5 ← dataset4/keyframe4.

It was captured using stereo endoscopic cameras mounted on a da Vinci Xi surgical robot. This is a high quality, high resolution dataset with smooth camera motions. Nonetheless, illumination of different sequences on this dataset varies considerably. We use images from the left camera rather than the stereo in this paper. As we either need to treat the stereo pairs as sequences or blend the pairs first, which may cause new uncertainties. We note this dataset includes camera motion measurements provided by robot kinematics, however, these are not accurate enough for moosaicking (see results in Fig. 4) and therefore we do not use this motion as a reference or groundtruth in this paper. The second is a fetoscopy placenta dataset (Bano et al., 2020) which has six sequences from different surgeries. Since this procedure is immersed in fluid, it does not contain specular reflections, but the scenes have very few discriminative textures and contain inconsistent motions due to floating particles crossing the field of view. By testing on this dataset, we want to verify generalisation of the proposed method in a significantly different environment and camera setting. The third dataset is a gastric endoscopy on a human cadaver using the Bellowscope gastroscope platform (Chandler et al., 2020). The environment is texture-less and with poor colour content, it has specular reflections, and has highly non-homogeneous illumination. Producing mosaics with this data represents the most extreme challenges for all studied algorithms. The numbers of frames of all the sequences of the three datasets are presented in Table 1. In this dataset, only small video sequences are tested. Due to the tubular shape of the anatomy, it does not make sense to build a single mosaic as the camera does a long trajectory through the digestive track, since it cannot be fully projected into a single plane without huge, non-intuitive distortions. No single algorithm works well in this setting. Instead, we do field of view expansion on localised portions of the anatomy where the endoscope is panning the scene.

We select three algorithms from the literature as comparison baselines, which correspond to mosaics as generated by the individual image registration approaches: feature-based (SIFT), direct pixel-based registration, and optical flow (FlowNet2.0). Each of them is a representative method in its own category (see Section 2). To further validate our approach, we also test our method when the covariance-weighted fusion is replaced with a naive simple average, and when loop closure is removed. In terms of quantitative analysis, we use the mosaicking metric described in Bano et al. (2019). This measures the structural similarity SSIM (Wang et al., 2004) between different overlapping mosaicking frames across the entire sequences. We compare frames with increasing temporal distance from 1 (i.e. all consecutive frames) up to 5 frames apart.

### 4.1 Results

The qualitative mosaicking comparison against the baselines on the SCARED, fetoscopy, and human cadaver datasets are presented, respectively, in Figs. 5, 6, and 7. We present the results using the naive fusion scheme (simple average) separately in Fig. 8 for all datasets. In terms of quantitative results, we display the boxplots of SSIM distributions in Fig. 9 for frame distances between 1 and 5. We highlight that, while our proposed method establishes effective (long) loop closure constraints, these only occur in 3 out of the 6 fetoscopy datasets, and in none of the SCARED and human cadaver sequences due to the simple nature of the camera motions in these cases. A comparison of our method with and without loop closures for a sequence of the fetoscopy dataset is displayed in Fig. 10. Finally, the average SSIM results for all reported methods, across all datasets is summarised in Table 2.

To understand the contribution of the different baseline algorithms to our fusion we also provide their indicative weights for each dataset. On the SCARED dataset, the traces of the information matrix have orders of magnitude $10^{5}$, $10^{4}$, and $10^{4}$ for the SIFT, direct registration, and optical flow respectively across the majority of image pairs. For the fetoscopy dataset, these values are $10^{2}$, $10^{5}$, and $10^{6}$ respectively. For the human cadaver dataset $10^{2}$, $10^{2}$, and $10^{4}$ for the first four sequences, and $10^{4}$, $10^{3}$, $10^{4}$ for the fifth sequence. In this context, higher relative values mean that our fusion scheme is giving more importance to the respective method.

**Table 2:** A comparison of different methods and ablation study on the three datasets. Values in the table are average SSIM with frame distance from 1 to 5. Note that the ablation study of loop closure is tested using the first three sequences of the fetoscopy dataset as there are long loop closures in these sequences.

Dataset	Seq.	SIFT	Direct reg.	Optical flow	Average fusion	Fusion w/o LC	Fusion (proposed)
SCARED	1	0.822	0.607	0.741	0.631	N/A	0.841
	2	0.804	0.526	0.674	0.540	N/A	0.807
	3	0.709	0.597	0.620	0.633	N/A	0.718
	4	0.677	0.549	0.628	0.527	N/A	0.680
	5	0.770	0.712	0.756	0.690	N/A	0.780

Fetoscopy	1	0.697	0.746	0.834	0.691	0.856	0.857
	2	0.850	0.820	0.852	0.845	0.862	0.873
	3	0.872	0.888	0.896	0.891	0.894	0.901
	4	0.907	0.905	0.924	0.914	N/A	0.924
	5	0.915	0.915	0.960	0.912	N/A	0.960
	6	0.917	0.912	0.917	0.917	N/A	0.926

Hum. cad.	1	0.641	0.659	0.662	0.645	N/A	0.730
	2	0.537	0.594	0.656	0.521	N/A	0.681
	3	0.501	0.501	0.638	0.534	N/A	0.641
	4	0.407	0.416	0.520	0.453	N/A	0.520
	5	0.596	0.577	0.641	0.638	N/A	0.645

### 4.2 Discussion

In the SCARED dataset (Fig. 5), SIFT has generally better performance than direct registration and optical flow, which can be explained by the high resolution and rich textures that make it easy to extract keypoint features. For sequences 3 to 5, direct registration fails entirely to work and most images in the mosaic overlap completely (i.e. the registration outputs a 3×3 identity matrix). However, SIFT fails to find good features on sequences 4 and 5, resulting in bad quality results. From the fourth column of Fig. 5, we can see that our fusion approach can remove errors of individual methods, relying on the ones with least covariance at any given point. In general, for this dataset, our fusion weights SIFT by an order of magnitude above the other two methods, which is consistent with the observed baseline performances. The quantitative results (Fig. 9) are also consistent with these results, showing our fusion having the best performance, closely followed by SIFT.

In the fetoscopy dataset (Fig. 6), the image resolution is not as high as that of the SCARED dataset. In addition, the environment is smooth and texture-less, which makes it difficult to extract keypoint features. Here, the SIFT-based mosaicking completely fails to work for all the six sequences (see the first column, the algorithm outputs a 3×3 identity matrix if there is not enough correspondences or inliers of the RANSAC method). The direct registration has a significant amount of drift, and the optical flow performs the best among the three baselines. From the last column of Fig. 6, we can see that the panorama generated by our proposed fusion performs the best. In general, our method provides lowest weights for SIFT estimations, and the highest to optical flow. The quantitative results in Fig. 9 also indicate that our fusion method produces in general higher SSIM scores, followed by optical flow.

In the human cadaver dataset (Fig. 7), the scene is mostly red and texture-less, which makes it very difficult to find correspondence or maximise similarity metrics. From sequence 1 to 4, both SIFT and direct registration fail to estimate the transformation between the images and cannot generate the panoramas. The optical flow-based mosaicking again performs the best out of the 3 baselines. In this dataset, our fusion also generally weights optical flow the highest. In fact, for the first four sequences, the fusion-based mosaicking results rely exclusively on the optical flow-based due to complete failure of other methods. For sequence 5, both SIFT and optical flow can generate a mosaic, but are not accurate in some regions (see the blue and green rectangles). The fusion-based method combines advantages of both the methods and gives a better panorama. For this challenging human cadaver dataset, the quality of the generated panorama is good with around 50 images using the proposed method.

The importance of weighting each method differently from frame-to-frame in our fusion approach is further validated by the fact that a simple average fusion works very poorly (see Fig. 8), which is further confirmed by the SSIM results in Fig. 9 and Table 2, where the average fusion is consistently close to the worst performers, since it is heavily contaminated by the worst of the three baselines at any given moment.

Finally, the effect of loop closure is the most significant on sequence 2 of the fetoscopy dataset (Fig. 10), where the camera performs a long trajectory before returning to the pre-visited area of the anatomy. Without loop closure, drift error is accumulated throughout the trajectory. When such a motion is not present (i.e. most of the other sequences), loop closure contributes little to the fusion performance.

All these experiments demonstrate the robustness of the proposed method and its generalisation across different datasets. Advantages of the proposed method over the state-of-the-art medical image mosaicking algorithms are validated through the comparison both with qualitative and quantitative results.

## 5 Conclusion

This paper presents a robust endoscopic image mosaicking framework based on fusion of multimodal estimation. One of the advantages of the proposed method is that it can work in different environments with no need to re-design the framework or finetune the parameters. Comparison with state-of-the-art baselines including SIFT-based, direct, end-to-end mosaicking shows that the proposed method is more robust to specular reflections or in feature-less environment. Moreover, the proposed framework is open to any other estimation method. It is rather straightforward to fit the new mosaicking methods into the proposed pose graph framework where only the evaluation of uncertainties of that method is needed. The limitations of the current framework include: It does not take the deformation into consideration; And it does not include the case that there may be outliers in the pose graph or inaccurate estimation of the uncertainties of the edges. In future work, we plan to solve these problems by developing an outlier-aware affine pose graph optimisation algorithm with deformation estimation.

## Declaration of Competing Interest

The authors declare that they have no known competing financial interests or personal relationships that could have appeared to influence the work reported in this paper.

## Data Availability

Data will be made available on request.
